# Limb remote ischemic conditioning of the recipient protects the liver in a rat model of arterialized orthotopic liver transplantation

**DOI:** 10.1371/journal.pone.0195507

**Published:** 2018-04-04

**Authors:** Zoltan Czigany, Christian Bleilevens, Christian Beckers, Christian Stoppe, Michaela Möhring, Andras Fülöp, Attila Szijarto, Georg Lurje, Ulf P. Neumann, René H. Tolba

**Affiliations:** 1 Institute for Laboratory Animal Science and Experimental Surgery, RWTH-Aachen University, Aachen, Germany; 2 Department of Surgery and Transplantation, RWTH-Aachen University, Aachen, Germany; 3 Department of Intensive Care Medicine, RWTH-Aachen University, Aachen, Germany; 4 HPB Research Center, 1st Department of Surgery, Semmelweis UniversityBudapest, Hungary; University of Colorado Denver, UNITED STATES

## Abstract

**Background:**

Ischemic-reperfusion (IR) injury still represents a major concern in clinical transplantation, especially in the era of extreme organ shortage and extended criteria donor organs. In the present study we aimed to investigate the hepatoprotective effects of remote ischemic conditioning (RIC) in a rat model of arterialized orthotopic liver transplantation (OLT).

**Methods:**

Male Lewis rats were used (n = 144 / 72 OLT cases; 240–340g) as donors and recipients. Livers were flushed and stored in 4°C HTK-solution for 8h before implantation. Recipients were randomly allocated into three experimental groups: RIC 1, RIC 2, Control. In RIC 1, RIC 2 groups, RIC was applied in the recipient before hepatectomy or after reperfusion (4x5-5min IR via clamping the infrarenal aorta), respectively. Animals were sacrificed at 1, 3, 24, 168h post-reperfusion (n = 6 recipient/group/time point). Hepatocellular injury, graft circulation, serum cytokines, tissue redox-stress and adenosine-triphosphate (ATP) levels have been assessed. Additional markers were analyzed, using Western blotting and reverse-transcription polymerase chain reaction.

**Results:**

RIC 1 group showed significantly (p<0.05) improved portal venous and microcirculation flow as well as velocity. RIC has significantly reduced tissue injury according to the serum levels of transaminases and results of histopathological evaluation. Reduced TUNEL-staining (p<0.01 RIC 1–2 vs. Control) and elevated pBAD/BAD ratio was detected in the RIC groups (p<0.01 RIC 1 vs. Control). Supporting findings were obtained from measurements of serum IL-10 as well as tissue malondialdehyde and ATP levels. Hemoxygenase-1 (HO-1) mRNA-expression was significantly higher in RIC 1 compared to Control (p<0.05 RIC 1 vs. Control).

**Conclusion:**

These results suggest that RIC might confer potent protection against the detrimental effects of IR injury including tissue damage, apoptosis, graft circulation, inflammation, tissue energetic status in OLT. HO-1 overexpression might play an orchestrating role in RIC mediated organ protection. An earlier intervention (RIC 1 protocol) was more effective than remote conditioning after graft reperfusion.

## Introduction

Due to the great improvements in surgical techniques, intensive therapy, organ preservation, and transplant immunology over the past decades, orthotopic liver transplantation (OLT) became the definitive treatment approach for end-stage liver diseases [1]. Ischemic-reperfusion (IR) injury, however, still represents a major risk factor for post-transplant functional graft impairment, acute- and chronic rejection, or for post-transplant hepatocellular carcinoma recurrence [2, 3]. These factors are even more important in clinical practice of the recent years, due to the extreme shortage of donor organs and the consequential need for alternative solutions (e.g. extended criteria donors, split liver transplantation, living donor liver transplantation, etc.) [3–6].

Since Toledo-Perayra et al. firstly demonstrated the presence of ischemic injury in transplanted canine livers in 1975 [7], several methods have been developed to reduce liver IR injury in different experimental and clinical settings.

The concept of remote ischemic conditioning (RIC) was introduced by Przyklenk et al., showing that brief ischemic reperfusion attacks, applied at a distant organ (e.g. limbs), can protect a certain target organ against the deleterious effects of IR injury via inducing robust innate cellular responses [8].

Whereas the RIC technique does reduce IR injury in different experimental models and clinical scenarios, the exact underlying molecular mechanisms and the definitive explanation of the phenomenon still remain unclear [9]. Although, our group has investigated and reported the effects of RIC in normothermic IR injury of the liver [10–12], the feasibility and efficacy of RIC applied in liver transplant recipients as well as the underlying mechanisms of this promising protective strategy remains to be elucidated [13]. In the past, various humoral and neural mechanisms of RIC have been depicted more or less as distinct pathways, however recent data suggested that the two are interdependent [14]. According to the currently prevailing hypothesis, RIC triggers afferent sensory nerves in the remote organ through local mediator release [9, 14]. Subsequently, this signal is transferred to the central nervous system and can modulate the firing of the vagal nerve. Vagal stimulation leads to secondary mediator release in various organs with autonomous innervation. These secondary mediators are transported to the target organ exerting the described protective effects via surface receptors and subcellular pathways [9, 14]. In a previous study we could show that denervation of the remote organ can completely abrogate the protective effects of RIC which is in line with findings in models of myocardial IR injury [15, 16]. Meanwhile others could demonstrate that plasma dialysate, containing essential humoral elements of RIC treated animals can protect isolated organs of an other non-treated animal against the deleterious effects of IR injury [14]. However, there are limited data available on exact mechanistic differences between the various mechanistic responses induced by remote conditioning before or after target organ ischemia (remote pre- vs. postconditioning) [16]. Therefore, in the present study we aimed to investigate whether the transplantation of a denervated organ into an otherwise neurologically intact recipient organism have an effect on the protective answer. This study was designed to investigate the effects of two different remote ischemic conditioning protocols on graft injury in an arterialized rat liver transplantation model. Numerous parameters, known to be involved in IRI and RIC, were used to assess local and systemic injury, protective responses following transplantation and RIC treatment.

## Materials and methods

The present study was designed according to the principles of the ARRIVE (Animal Research: Reporting of *In Viv*o Experiments) guidelines and based on our review article on performing and reporting experimental studies in rat liver transplantation [17].

### Animals

Experiments were performed in accordance with the institutional guidelines and the German federal law regarding the protection of animals. The full ethical proposal was approved by the responsible authorities (LANUV NRW–“Landesamt für Natur, Umwelt und Verbraucherschutz Nordrhein-Westfalen”, Recklinghausen, Germany, 84–02.04.2014.A032). All animals in the present study received human care according to the principles of the “Guide for the Care and Use of Laboratory Animals” (8^th^ edition, NIH Publication, 2011, USA).

Male Lewis rats (LEW/OrlRj; Janvier Labs, Le Genest Saint Isle, France) were used as donors and recipients in a model of isogenic liver transplantation (Σn = 144 / 72 OLT cases; median bodyweight: 278 g, range: 240–340 g). Bodyweight difference between donor and recipient pairs was ≤10 g. The animals were housed under specific pathogen-free conditions according to the guidelines of the “Federation for Laboratory Animal Science Associations” (FELASA; www.felasa.eu) in a temperature- and humidity-controlled barrier environment with a 12-h light and dark cycle. Standard pellets for laboratory rats (Sniff GmbH, Soest, Germany) and water were granted *ad libitum*.

### Surgical technique

All experiments were performed at the same time of day to avoid disturbing effects of circadian rhythm. Animals were anesthetized using 2vol% isoflurane (Forane; Abbott GmbH, Wiesbaden, Germany) during all the surgical interventions. All surgical procedures were performed by a single surgeon (ZC). Surgical techniques of arterialized OLT in rats were described by our team in a technical video publication [18]. Briefly, the donor liver was prepared for graft retrieval. Following satisfactory mobilization of the liver and the main vessels, 500 international units (IU) of heparin-natrium (Heparin-Natrium-ratiopharm; Ratiopharm GmbH, Ulm, Germany) in 2 mL of Ringer solution was injected via the penile vein. After 2 minutes, the liver was perfused via the portal vein with 60 mL of 4°C HTK (Histidine-tryptophan-ketoglutarate) solution (Custodiol; Dr Franz Köhler Chemie GmbH, Bensheim, Germany) at a hydrostatic pressure of 20 cm H2O. Back table preparation was performed according to Nagai et al. [18]. The liver graft was then stored in HTK solution with a target cold ischemic time (CIT) of 8 hours at 4°C using an external computer controlled cooling circuit (Ministat 125; Peter Huber Kältemaschinenbau GmbH, Offenburg, Germany).

**Fig 1:**
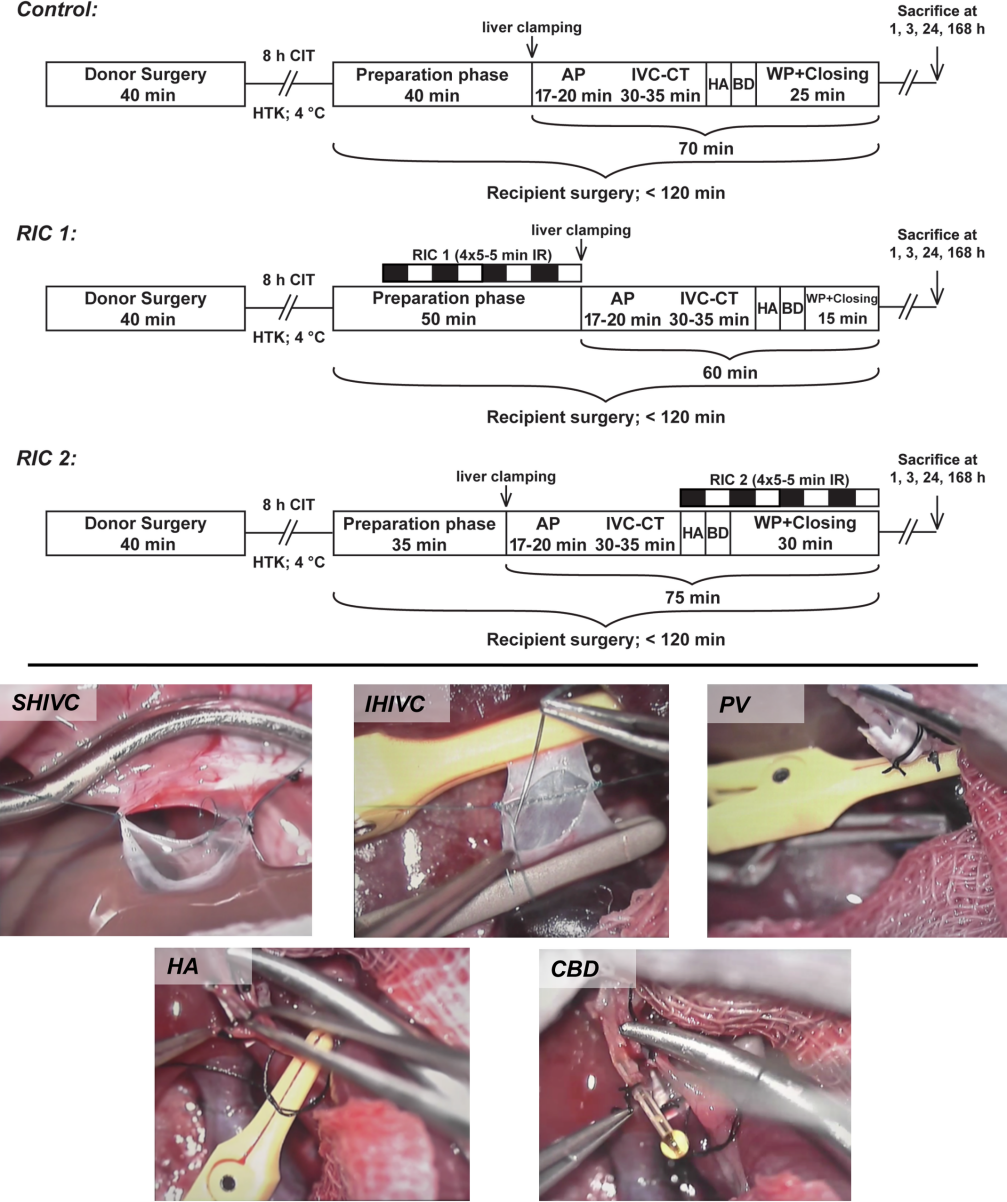
Study flowchart and microsurgical technique. Animals were randomized into three experimental groups (Control, RIC 1, RIC 2). Following liver retrieval, grafts were stored for 8 h in cold HTK solution and implanted into the recipient animals. In the RIC 1 and RIC 2 groups, remote conditioning (4x5 min IR via exclusion of the infrarenal aorta) was applied before the anhepatic phase or after reperfusion, respectively. Short waiting periods have been implemented to keep the recipient procedures in the same time range in all experimental groups. Animals were sacrificed after 1, 3, 24, and 168 h of reperfusion for sample collection and further analysis (n = 6/group/time point). Representative photographs are demonstrating the used microsurgical approach for liver transplantation. Original magnification 8-16x. SHIVC: 7–0 Prolene one-way up running suture; IHIVC: 8–0 Prolene one-way up running suture; PV: Cuff technique, using 14 G cuffs; CBD and HA: Splinting with 24 G polyethylene splints. Abbreviations used: CIT-cold ischemic time; HTK-Histidine-tryptophan-ketoglutarate solution; AP-anhepatic phase; IVC-CT-inferior vena cava clamping time; HA-hepatic artery; CBD-common bile duct; WP-waiting period; RIC-remote ischemic conditioning; IR-ischemia reperfusion; SHIVC-supra hepatic inferior vena cava; IHIVC-infra hepatic inferior vena cava; PV-portal vein.

A second animal has been prepared to receive the liver graft. Following recipient hepatectomy, the liver graft was placed in an orthotopic position in the abdominal cavity. End-to-end reconstruction of the suprahepatic inferior vena cava was performed using continuous sutures (Prolene 7–0; Ethicon, Somerville, NJ, USA). The portal vein was anastomosed using a 3.5 mm long 14-gauge polyethylene cuff with circumferential grooves (Vasofix 14G; B. Braun). Subsequently the graft was reperfused. Infrahepatic inferior vena cava was then reconstructed in an end-to-end fashion using continuous sutures (Prolene 8–0; Ethicon). For graft re-arterialization and common bile duct reconstruction 24-gauge polyethylene stents (Vasofix 24G; B. Braun) were used in a length of 3.5 mm and 5 mm, respectively. For details of surgical techniques see representative intraoperative photos (Fig 1). Fluid resuscitation and fluid administration protocols were described in detail previously [18].

Remote ischemic conditioning treatment was applied only in the recipient animals as 4 cycles of 5 min of ischemia and 5 min of reperfusion (40 min) by clamping of the infrarenal aorta using an atraumatic microvascular clamp (Aesculap Yasargil FT260T; B.Braun) as described previously [10] (Fig 1). At the end of the surgical procedure, the abdominal incision (in 3, 24, 168 hours of reperfusion groups) was closed in two layers using continuous 4–0 sutures (Vicryl 4–0; Ethicon).

Postoperatively, recipient rats were placed in a special intensive care unit cage (Vetario; Brinsea Products Ltd, North Somerset, United Kingdom) for a 60 min long recovery period with warmed air (30–35°C) and an oxygen supply. Antibiotic treatment and analgesia were achieved by subcutaneous injections of cefuroxime sodium (16 mg/kg/24 h) (Cefuroxim Fresenius; Fresenius Kabi Deutschland GmbH, Bad Homburg, Germany) and buprenorphine (0.03 mg/kg/24 h) (Temgesic; EssexPharma, Haar, Germany) for 72 h after surgery. During the first 4 hours postoperatively, animals were observed continuously and then transferred back to their normal environment. During the first 3 postoperative days all animals were visited at least every 12 hours by the surgeon and/or by an experienced veterinary technician and scored using a study specific severity assessment and human end-points score-sheet including factors such as body-weight changes, general state of the animals, spontaneous behavior, clinical parameters (temperature, respiration, peripheral circulation). In the subsequent follow-up, animals have been visited at least once a day until sacrifice. Following the corresponding observation periods samples were collected and animals were subsequently sacrificed under deep isoflurane anesthesia 2vol%-4vol% and buprenorphine (0.03 mg/kg) analgesia.

### Experimental design

For the present study 72 cases of arterialized whole-graft rat OLTs were performed based on an *a priori* sample size estimation. Recipients were randomly allocated into three experimental groups (n = 24 cases/group) (Fig 1).

Control: no remote conditioning was applied.

RIC1: remote ischemic conditioning protocol was applied before liver exclusion and recipient hepatectomy.

RIC2: remote ischemic conditioning protocol was applied after graft reperfusion (reperfusion of the IHIVC).

After 1, 3, 24, and 168 hours of portal reperfusion, graft microcirculation as well as portal flow and pressure were measured in anesthesia (n = 6 cases/group/time point). Systemic venous blood from the vena cava and tissue samples were collected for analysis before the animals were sacrificed via exsanguination in deep anesthesia. Fig 1. depicts a flowchart of the experimental protocol.

### Graft microcirculation and portal venous circulation

Graft microcirculation and red blood cell velocity were measured using an O2C device with a corresponding surface probe (O2C-oxygen to see device, LF1 surface probe; LEA Medizintechnik GmbH, Giessen, Germany). Mean of the measurements from 4 standard points on the liver surface were used to characterize graft microcirculation.

Transit-time perivascular flowmeter was used for portal venous flow measurements (T403 device, MA2PSB flow probe; Transonic Systems, Inc., Ithaca, NY, USA). Portal venous pressure was measured using a monitoring device (Sirecust 404; Siemens, Erlangen, Germany) following direct puncture of the portal vein with a 27-gauge needle (BD Microlance 3; Becton Dickinson GmbH, Heidelberg, Germany).

### Biochemical analysis and serum cytokines

Blood samples, collected from the inferior vena cava at sacrifice, were centrifuged (4°C, 10 min, 2500 rpm) and then serum levels of alanine aminotransferase (ALT), aspartate aminotransferase (AST), lactate dehydrogenase (LDH), and total bilirubin levels were measured using standard photometric procedures in an automated analyzer (Vitros 250; Johnson and Johnson, Neuss, Germany).

Serum samples, stored at -80°C, were used for interleukin-10 (IL-10), monocyte chemoatractant protein-1 (MCP-1) assessments at 1, 3, and 24 hours of reperfusion using commercial rat enzyme-linked immunosorbent assay (ELISA) kits (R and D Systems, Minneapolis, MN, USA) according to the manufacturer’s instructions.

### Lipid peroxidation and liver tissue adenosine triphosphate concentration

Free radical stress was assessed via measuring malondialdehyde (MDA) concentrations using fluorescence spectrophotometry (Tecan Infinite; Tecan Deutschland GmbH, Crailsheim, Germany) as described in detail previously [19, 20].

One portion of the left lateral lobe was snap-frozen with liquid nitrogen pre-cooled metal tongs, and then stored at -80°C until the assessment of liver tissue adenosine triphosphate (ATP) concentrations, as described in detail elsewhere [5, 21].

### Histopathology and TUNEL immunohistochemistry

Histological samples were harvested from identical anatomical sites (mediate lobe of the liver). The excised liver was fixed in 10% neutral buffered formalin and embedded in paraffin. Slides, 4–6 μm thick, were stained with hematoxylin and eosin (HE). The examining pathologists was not informed regarding the applied treatment or grouping. Slides were all examined in a blinded fashion by two independent investigators, including a senior veterinary pathologist (M.M.). The used semi-quantitative scoring system was modified from the previous scoring of Yagi et al. [19]. Histological signs of injury (hepatocyte vacuolization, hepatocyte degeneration, tissue necrosis, tissue hemorrhage, neutrophil infiltration) were graded individually on a scale from 1 to 4 (1 = no changes or negligible lesions, affecting 0–10% of the field; 2 = mild, lesions affecting 10%–40% of the field; 3 = moderate, lesions affecting 40%–70% of the field; 4 = severe, lesions affecting >70% of the field). Ten randomly chosen, non-overlapping fields (400X magnification) were evaluated with light microscopy (Leica DM 2500; Leica Microsystems GmbH, Wetzlar, Germany). To simplify this complex scoring, a total score, the sum of the aforementioned five individual parameters with a maximum of 20 points/animal, was introduced. This total score was used when presenting the results.

Terminal deoxynucleotidyl transferase-mediated dUTP nick end labeling (TUNEL) immunohistochemistry was performed using commercial staining kits (Apoptag; ICHEMICON, Schwalbach/Ts, Germany) as described previously [19]. Ten randomly chosen high-power fields (x400) were selected, without significant necrotic regions, for counting TUNEL positive cells. Cells were considered apoptotic when, besides TUNEL positivity, morphological signs of apoptosis were also present [22].

### mRNA expression by reverse-transcription polymerase chain reaction

For assessment of alterations in liver tissue messenger ribonucleic-acid (mRNA) expression, Reverse-Transcription Polymerase Chain Reaction (RT-PCR) was performed using TaqMan technology, as described previously [5]. TaqMan probes and primers for hemoxygenase-1 (HO-1) were used (Applied Biosystems, Life Technologies Japan Ltd., Japan).

### Protein expression by Western blotting

For Western blotting liver tissue was homogenized with ice-cold lysis buffer (Sigma-Aldrich, Germany) completed by Protease inhibitor cocktail tablets (Roche Diagnostics, Mannheim, Germany). Protein concentration in supernatants was determined (DC-Protein Assay Kit, BIO-RAD Laboratories, Munich, Germany). Proteins were separated by 10% SDS-Page, and transferred onto a PVDF membrane (BIO-RAD), according to a standard semi-dry blotting procedure (60min, 25V). Unspecific binding-sites were blocked. Incubation with specific antibodies against phosphorylated- and total regulator protein Bcl-2-associated death promoter (BAD) and Glyceraldehyde 3-phosphate dehydrogenase (GAPDH) as housekeeper (phosphoBAD: #5284, BAD: #9239, GAPDH: #5174, all from Cell Signaling Technology, Danvers MA, USA) was performed. Incubation was followed by repeated washing steps (3x5 min in TBS buffer containing 1% Tween20; Sigma Aldrich), prior to the incubation with a horseraddish-peroxidase conjugated goat anti-rabbit antibody (#7074; Cell Signaling Technology) for 1h at room temperature on a shaker. The final reaction was visualized by enhanced chemiluminescence using an imaging system (Clarity WesternECL Blotting Substrate, ChemiDoc MP System, BIO-RAD), and the images were analyzed densitometrically using Image Lab Software (BIO-RAD). The results were displayed as integrated density value (IDV), relative to GAPDH.

### Statistical analysis

Results are expressed as mean ± standard deviation (S.D.) for each group, with the exception of the histological scores, where median and inter-quartile ranges (IQR) are reported. Two-way analysis of variance (ANOVA) and Bonferroni post-hoc correction was performed to analyze changes in time dependent parameters and between group differences in each time point. One-way ANOVA was used to test the differences within three groups. For analysis of histological scores, non-parametric Kruskal-Wallis H test was applied. Differences were considered significant when p < 0.05. Calculations and data plotting were performed using IBM SPSS v24 (IBM Inc., Armonk, NY, USA) and GraphPad Prism 7 (GraphPad Software Inc., San Diego, CA, USA) software packages.

## Results

No significant differences were found between groups, concerning CIT (479.1±4.2 min for all groups; p = 0.36 between groups, one-way ANOVA) and anhepatic time (18.3±0.8 min for all groups; p = 0.12 between groups, one-way ANOVA). One-week survival was 100% in each group (6/6 recipients survived/experimental group).

### Graft microcirculation and portal venous circulation

**Fig 2:**
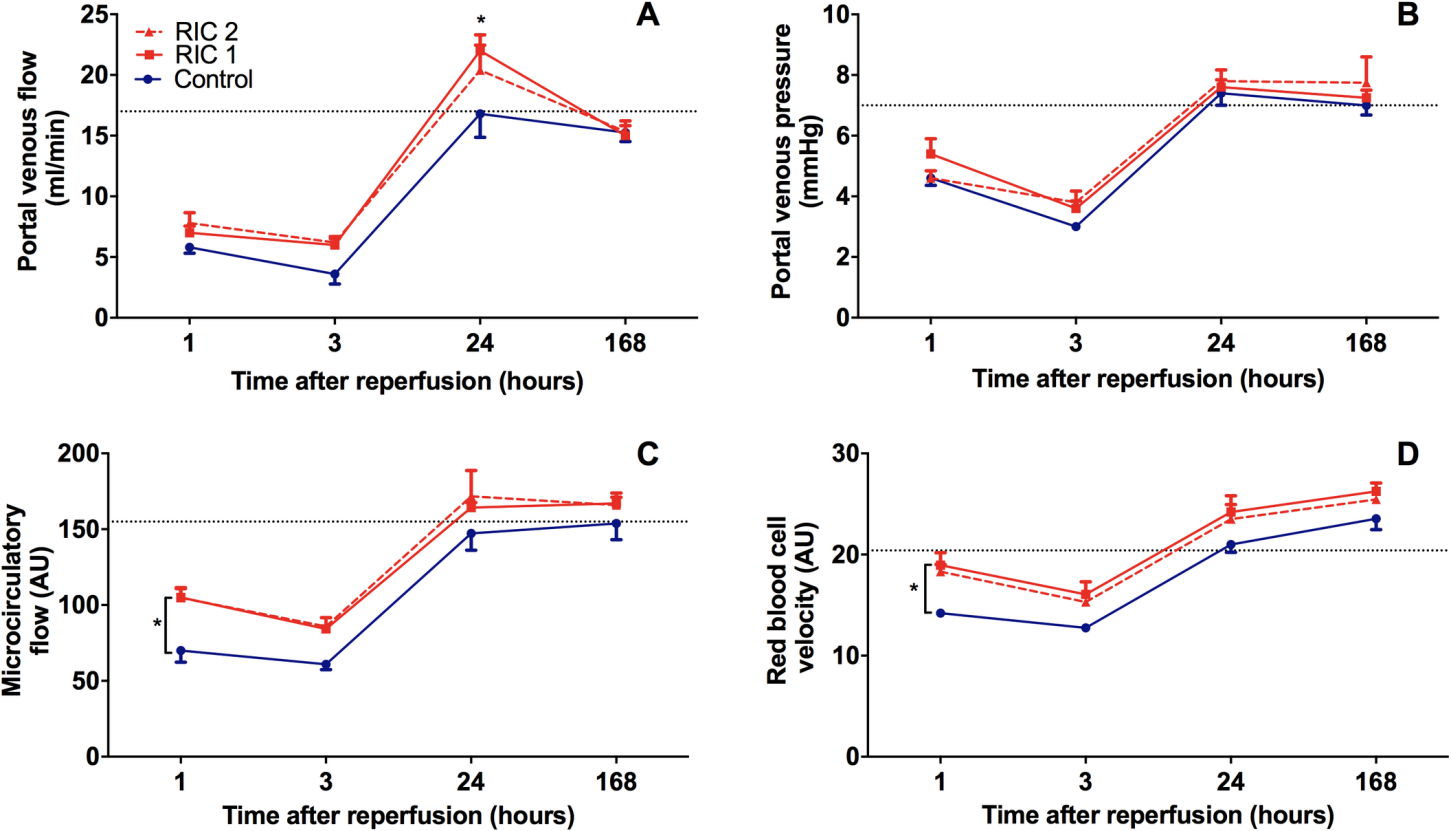
Liver graft macro and microcirculation. (A; B) Time course of portal venous flow and pressure. Portal venous flow was significantly higher in the RIC 1 group compared to Control after 24 hours of reperfusion (mean±SD, *p<0.05 RIC 1 vs Control, two-way ANOVA and Bonferroni post-hoc test, n≥5/group/time point). No significant differences have been found in portal pressure. (C; D) Graft microcirculatory parameters measured with the O2C device, such as flow and velocity, remained higher in the RIC group compared to Control throughout the observation period. Microcirculation was significantly higher after 1 hour of reperfusion in the RIC 1 group vs. Control (mean±SD, *p<0.05 RIC 1 vs. Control, two-way ANOVA and Bonferroni post-hoc test, n = 6/group/time point). Similar trends but with marginally non-significant differences were found in the RIC 2 group. Dotted line = baseline values after laparotomy in healthy animals (n = 15). Abbreviations used: AU-arbitrary unit, O2C-oxygen to see.

During the reperfusion period no considerable differences were found in the characteristics of the microcirculatory parameters between groups (Fig 2). Following an initial decrease in microcirculation and red blood cell velocity the values have returned to the normal levels at 24 hours in each group (Fig 2). Treated groups (RIC 1 and RIC 2) showed slightly improved microcirculation compared with the Control group throughout the observation period (Fig 2). At the 1^st^ hour of reperfusion a significant difference was detected between Control and RIC 1 groups in liver microcirculation and red blood cell velocity (RIC 1_1hour_ vs. Control_1hour_, 105.1±13.1 vs. 70.1±17.3 AU, p = 0.049 // RIC 1_1hour_ vs. Control_1hour_, 19.0±1.2 vs. 14.2±0.4 AU, p = 0.014, respectively). There was a marginal positive trend regarding microcirculation and velocity between RIC 2_1hour_ and Control_1hour_ groups which did not reach, however, the level of statistical significance (Fig 2, RIC 2_1hour_ vs. Control_1hour_, p = 0.053 and p = 0.053, respectively).

After a prominent drop in all experimental groups, portal venous pressure and flow have returned approximately to the normal level by 24 hours (Fig 2). Significantly improved portal venous flow was observed in RIC 1_24hours_ group when compared with the Control_24hours_ group (RIC 1_24hours_ vs. Control_24hours_, 22.0±2.9 vs. 16.8±4.3 ml/min, p = 0.019). No significant differences were found in portal pressure during the experiments (Fig 2).

### Biochemical analysis and serum cytokines

**Fig 3:**
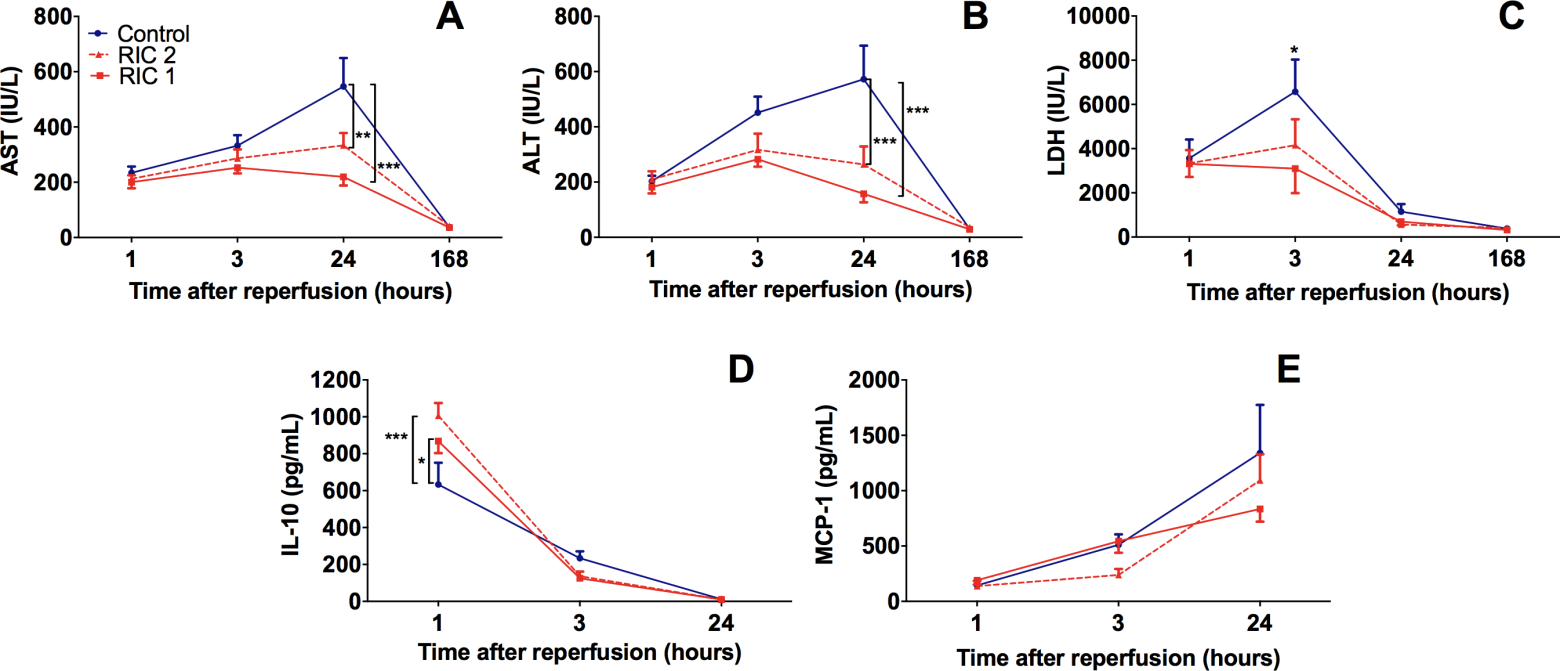
Hepatocellular injury and inflammatory cytokines. (A; B; C) Time course of transaminases and LDH demonstrated the peak of hepatocellular injury after 24 and 3 h, respectively. The application of RIC significantly reduced transaminase and LDH release (mean±SD, *p<0.05, **p < 0.01,***p < 0.001 RIC 1 and RIC 2 vs. Control two-way ANOVA and Bonferroni post-hoc test, n = 6/group/time point). (D; E) Serum IL-10 was significantly increased in the early phase or reperfusion (1 h). The RIC 1 and RIC 2 protocols resulted in a further increment in the serum release of this anti-inflammatory master-cytokine. (mean±SD, *p<0.05, ***p<0.001 RIC 1 and RIC 2 vs. Control, respectively two-way ANOVA and Bonferroni post-hoc test, n = 6/group/time point). Levels of IL-10 decreased below threshold for detection after 24 hours. MCP-1 showed reciprocal characteristics, however, without significant between group differences. Abbreviations used: AST-aspartate aminotransferase; ALT-alanine aminotransferase; LDH-lactate dehydrogenase; IL-interleukin; MCP-Monocyte chemoattractant protein, RIC-remote ischemic conditioning.

Hepatocellular damage, monitored by the measurement of serum ALT and AST levels, has increased prominently with a peak on the 1^st^ postoperative day in the Control group (Fig 3). After 24 hours of reperfusion, remote conditioning treatment could potently reduce ALT and AST levels compared with the Control_24hours_ group (ALT: RIC 1_24hours_ vs. Control_24hours_, 157.6±69.5 vs. 572.6±271.7 IU/l; RIC 2_24hours_ vs. Control_24hours_, 263.8±145.1 vs. 572.6±271.7, p<0.001, p<0.001, respectively // AST: RIC 1_24hours_ vs. Control_24hours_, 219.4±70.4 vs. 546.8±230.3 IU/l; RIC 2_24hours_ vs. Control_24hours_, 333.4±99.9 vs. 546.8±230.3, p<0.001, p<0.01, respectively). Concerning LDH, an earlier peak was observed in each experimental group after 3 hours of graft reperfusion. RIC 1_3hours_ had significantly lower levels of serum LDH (p = 0.018) when compared with the Control_3hours_ group (Fig 3). ALT, AST, and LDH values have declined to normal values by 168 hours in all experimental groups (Fig 3.). Despite the slight differences, observed graphically, between the two RIC groups, no statistical significance could be shown regarding the AST, ALT and LDH values after either reperfusion period (Fig 3.).

No pathological elevation was observed in total bilirubin levels measured after 24 and 168 hours of reperfusion (values stayed under 0.3 mg/dL in each experimental group throughout the experiments, therefore no further analysis and graphical presentation were performed).

Serum levels of the anti-inflammatory cytokine, IL-10, have peaked after 1 hour of reperfusion (Fig 3). Significantly higher IL-10 levels were detected in the RIC 1_1hour_ and RIC 2_1hour_ groups compared to the Control_1hour_ group (RIC 1_1hour_ vs. Control_1hour_, 868.4±159.3 vs. 632.9±289.9, p = 0.042; RIC 2_1hour_ vs. Control_1hour_, 1006.3±168.6 vs. 632.9±289.9, p<0.001) With the course of reperfusion a prominent reduction was observed in IL-10 concentration in each experimental group (Fig 3).

Pro-inflammatory cytokine, MCP-1 level showed reciprocal characteristic features. MCP-1 levels peaked at 24 hours of reperfusion, however, without significant differences between experimental groups (Fig 3).

### Histopathology

**Fig 4:**
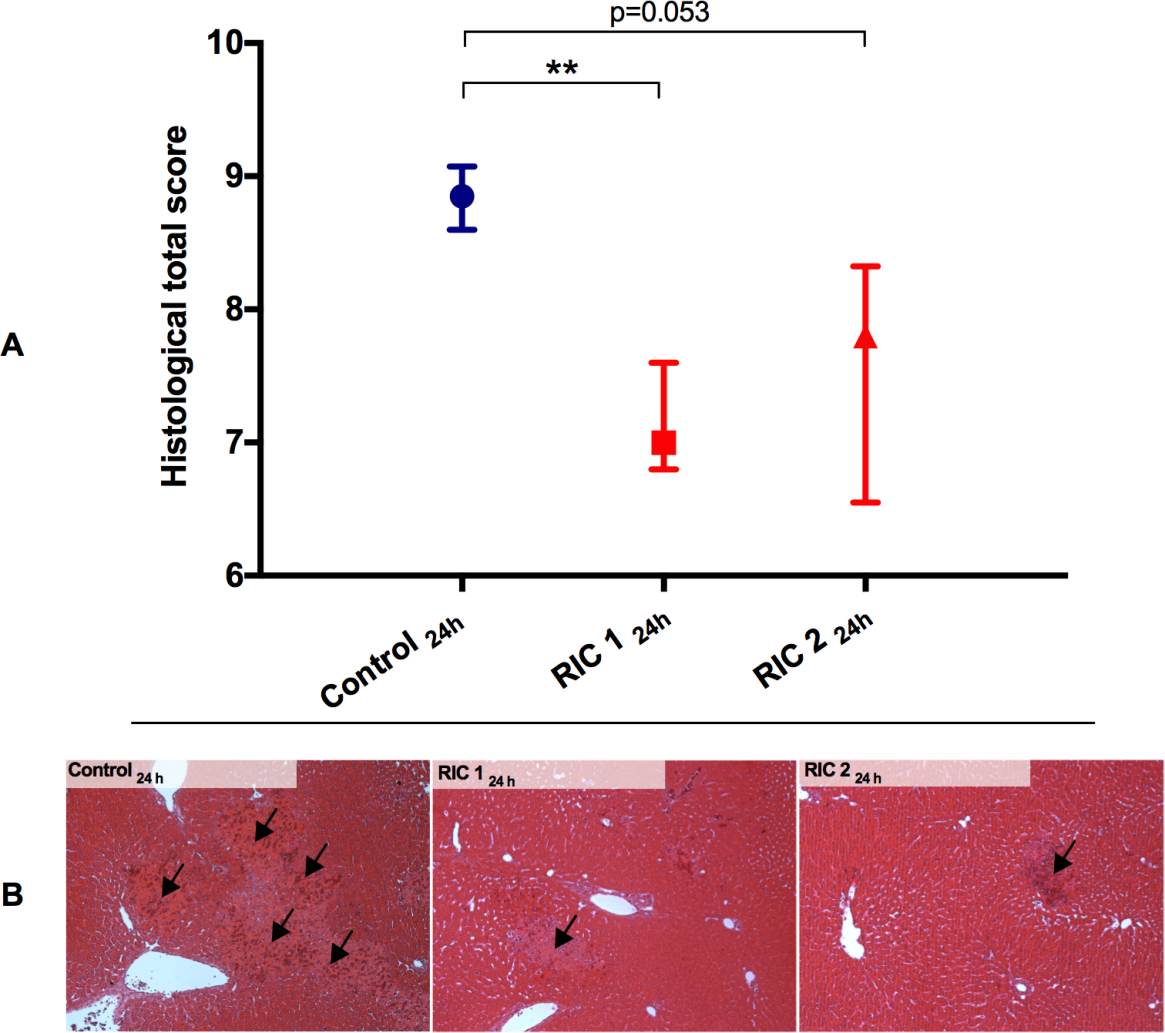
Histopathological injury. (A) Morphological damage peaked after 24 hours of reperfusion. Semi-quantitative scoring resulted in a markedly reduced total score in the RIC groups vs. Control (mean±IQR, **p<0.01 RIC 1 vs. Control, Kruskal-Wallis H Test, n = 6/group/time point). (B) Representative photos of the histological specimens following transplantation and 24 h reperfusion (stained with hematoxylin-eosin; original magnification 100x). Mild-moderate grade hepatocyte degeneration, vacuolization, tissue necrosis and hemorrhage as well as inflammation were observed on the slides of all the three experimental groups; nevertheless, a milder injury was seen on the slides of the RIC 1 and 2 groups (arrows). Abbreviations used: RIC-remote ischemic conditioning.

Morphological damage reached its peak by 24 hours of reperfusion according to the semi-quantitative histological scores (Fig 4). Mild-moderate grade hepatocyte degeneration, vacuolization, tissue necrosis and hemorrhage as well as inflammation were observed on the slides of all the three experimental groups. Nevertheless, significantly reduced total score values were found in the RIC 1 compared with the Control group (RIC 1_24hours_ vs. Control_24hours_, median: 7.0 IQR: 6.8–7.6 vs. median: 8.9 IQR: 8.6–9.1, p = 0.006; RIC 2_24hours_ vs. Control_24hours_, median: 7.8 IQR: 6.6–8.3 vs. median: 8.9 IQR: 8.0–9.3, p = 0.053). Fig 4 shows the representative pathological findings from the evaluation of samples stained with HE 24 h after reperfusion. After 168 h regenerative processes were dominating without any specific between group differences.

### Tissue ATP concentration and lipid peroxidation

**Fig 5:**
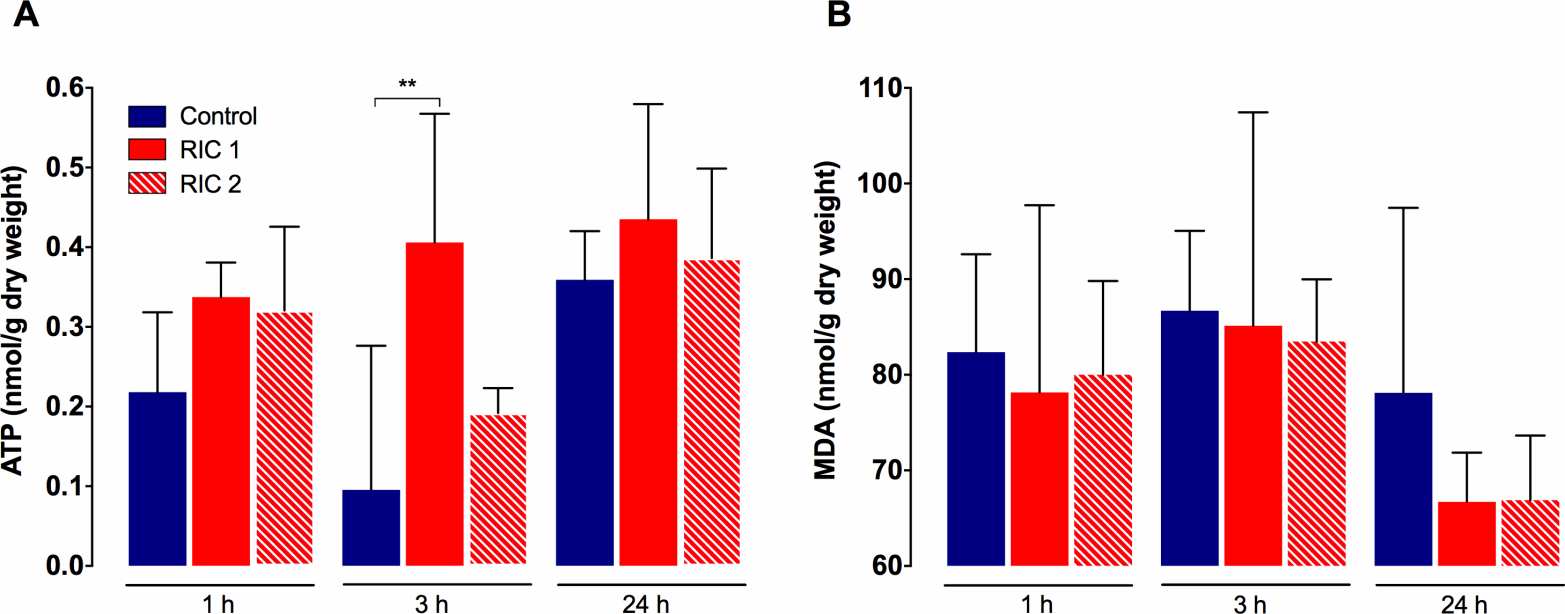
Liver tissue ATP levels and lipid peroxidation. (A) RIC resulted in better preserved tissue ATP levels throughout the observation period. Significantly higher tissue ATP levels have been found in the RIC 1 group vs. Control after 3 h reperfusion. (mean±SD, **p<0.01 RIC 1 vs. Control, one-way ANOVA, n = 6/group/time point). (B) Extent of oxidative damage and lipid peroxidation (MDA) did not show significant between group differences. Abbreviations used: RIC-remote ischemic conditioning; ATP-adenosine triphosphate; MDA-malondialdehyde.

In Control and RIC 2 groups similar characteristic features were observed concerning tissue ATP levels (Fig 5), meanwhile the RIC 1 group showed a more preserved energetic status during the experiment. There was a significant difference between RIC 1 and Control groups after 3 hours of reperfusion (RIC 1_3hours_ vs. Control_3hours_, 0.41±0.16 vs. 0.10±0.18 mmol/g dry weight, p = 0.002). No significant disparity could be found between the RIC 2 and Control or the two remote conditioning groups (Fig 5).

Liver tissue MDA levels, measured after 1, 3, and 24 hours of reperfusion, were lower in the RIC 1 and RIC 2 groups throughout the whole observation period (Fig 5). Nevertheless, no significant between group differences could be seen after either of the registered time-points (Fig 5).

### Liver tissue HO-1 mRNA

**Fig 6:**
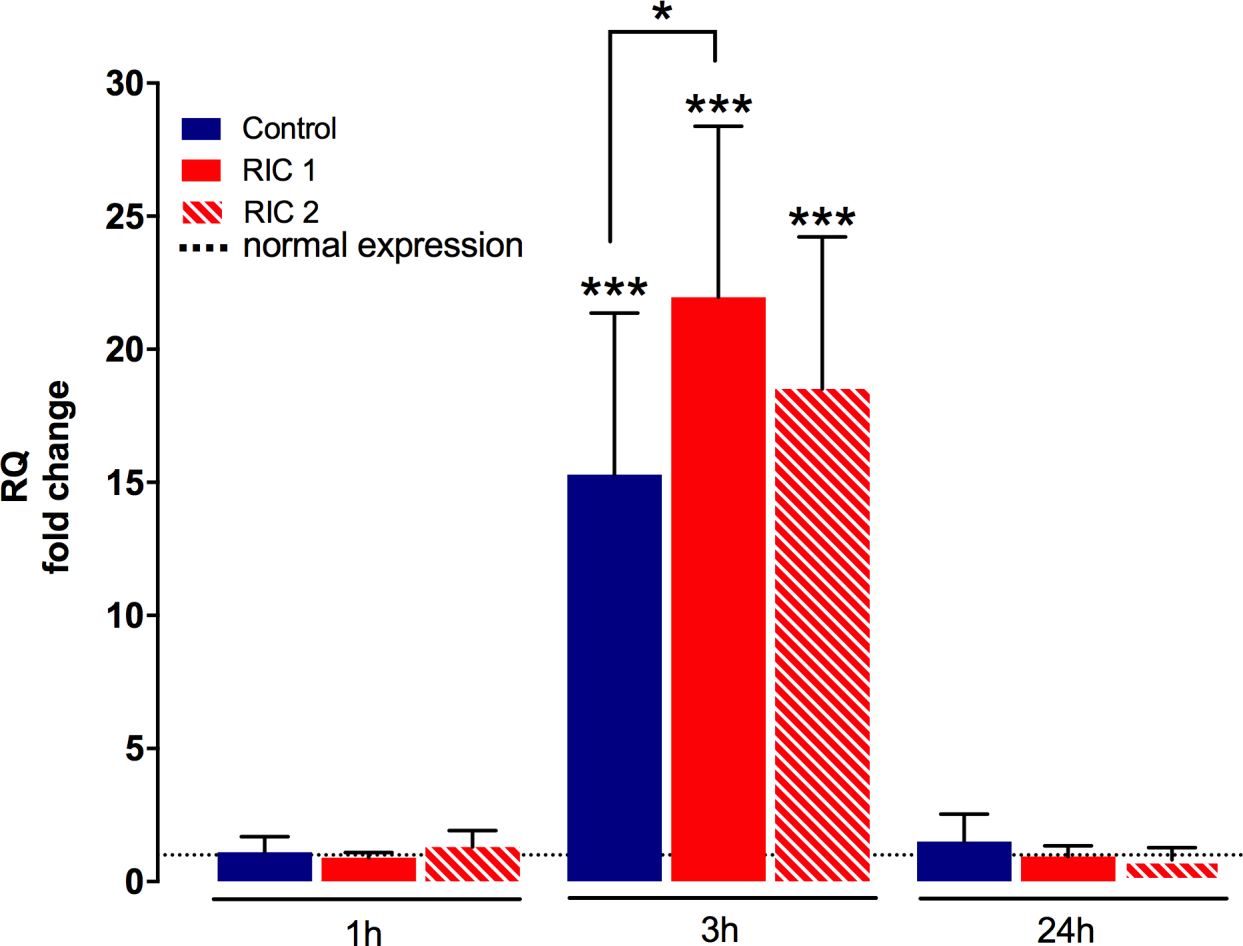
Liver tissue HO-1 encoding mRNA. mRNA expression of HO-1 increased markedly after 3 hours of reperfusion compared to the physiological levels (mean±SD, ***p<0.001 Experimental groups vs. Healthy control, one-way ANOVA, n = 6/group/time point). RIC treatment could further increase HO-1 expression resulting in a significant difference between RIC 1 group vs. Control. (mean±SD, *p<0.05 RIC 1 vs. Control, one-way ANOVA, n = 6/group/time point). Dotted line = physiological values in healthy animals. Abbreviations used: RQ-relative quantification; RIC-remote ischemic conditioning; HO-1-heme oxygenase-1.

Tissue encoding mRNA expression of HO-1 showed a strong elevation compared to the physiological HO-1 levels after 3 hours of reperfusion (p<0.001 vs. normal expression measured in healthy rat livers, Fig 6). Significantly higher HO-1-mRNA expression was detected in the RIC 1_3hours_ group when compared to the Control_3hours_ group (RIC 1_3hours_ vs. Control_3hours_, 21.95±6.43 vs. 15.30±6.07 RQ fold change, p = 0.04).

### Liver tissue pBAD/BAD and TUNEL immunohistochemistry

**Fig 7:**
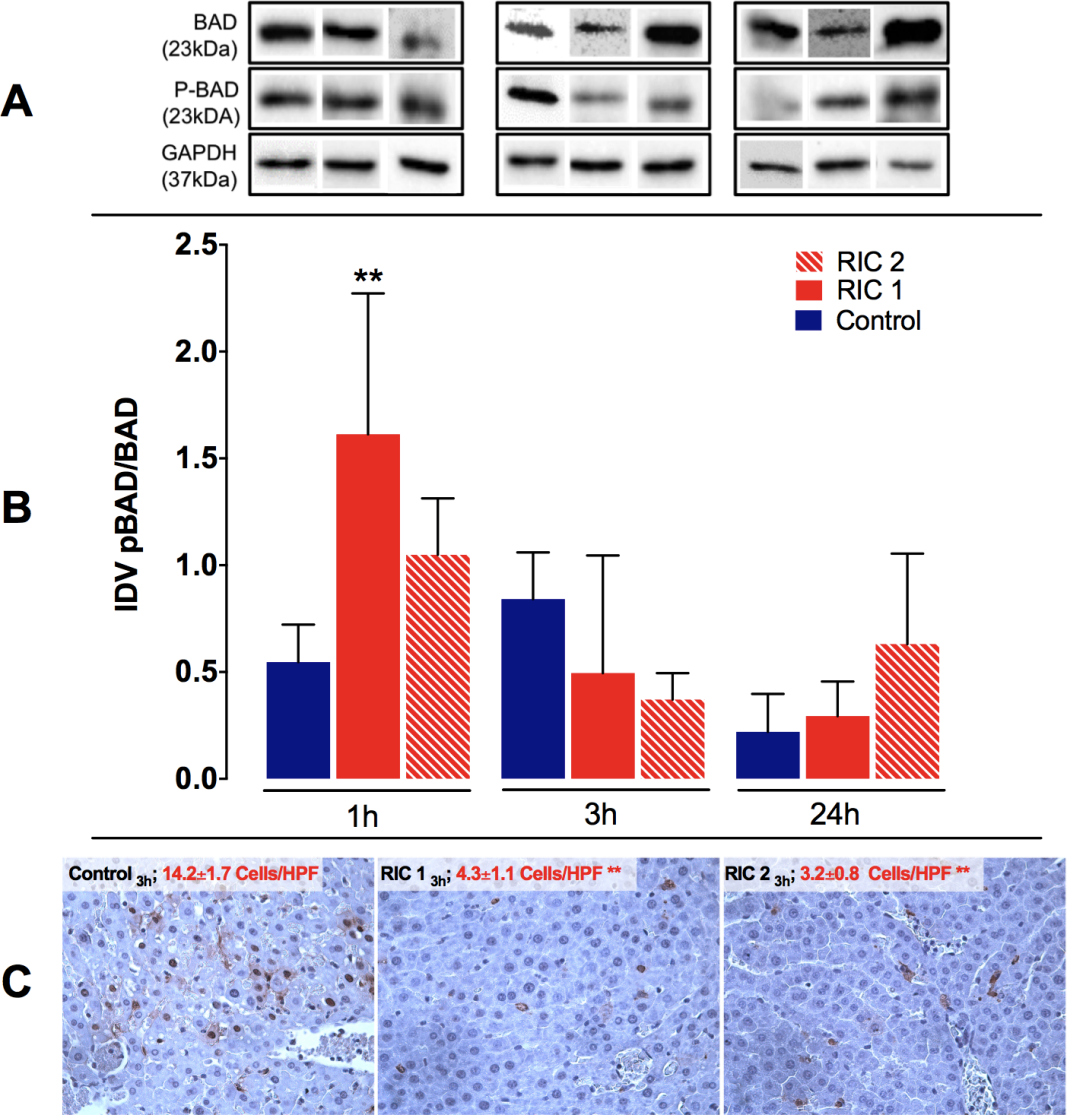
Liver tissue pBAD/BAD ratio and TUNEL immunohistochemistry. (A) Representative photos of pBAD/BAD expression for all groups following SDS-PAGE and WB. (B) pBAD/BAD ratio was significantly increased in the RIC 1 group vs. Control after 1 h of reperfusion (mean±SD, **p<0.01 RIC 1 vs. Control, one-way ANOVA, n = 6/group/time point). (C) Representative photos of histological slides stained with TUNEL immunohistochemistry. A significant reduction in TUNEL positive apoptotic cells was found following RIC treatment, liver transplantation and 3 hours of reperfusion (mean±SD, **p<0.01 RIC 1 and RIC 2 vs. Control, one-way ANOVA, n = 6/group/time point). Abbreviations used: SDS-PAGE-sodium dodecyl sulphate-polyacrylamide gel electrophoresis; IDV-integrated density value; HPF-high power field; RIC-remote ischemic conditioning.

Liver tissue protein expression ratio of pBAD/BAD showed a significant increase after 1 hour of reperfusion in the RIC 1_1hour_ group compared to the Control_1hour_ group (RIC 1_1hour_ vs. Control_1hour_, 1.61±0.66 vs. 0.55±1.55 IDV, p<0.01). In the later phase of reperfusion unspecific changes were observed in pBAD/BAD ratio without significant between group differences (Fig 7).

Concerning TUNEL immunohistochemistry staining, there was a significant difference between RIC treated groups and the Control group in numbers of apoptotic cells after 3 hours of liver reperfusion (RIC 1_3hours_ and RIC 2_3hours_ vs. Control_3hours_, 4.3±1.1 and 3.2±0.8 vs. 14.2±1.7 cells/HPF, p<0.01, respectively) (Fig 7). At the next observation time point (24 hours) the extended TUNEL positive, degenerative and necrotic regions were dominant on the slides (correlating well with the observations made during the evaluation of the HE stained samples); meanwhile, the number of TUNEL positive cells also showing the morphological signs of apoptosis was reduced.

## Discussion

The effects of remote ischemic conditioning have been investigated in IR scenarios of different organs (e.g. myocardium [23, 24], brain [25], kidney [26, 27], and by our group on liver [10, 12]) in multiple experimental and clinical studies over the past years [9]. However, available and consistent datasets from well-designed and comprehensive experimental studies, which would demonstrate the effects of this approach in liver transplantation are rare (Table 1.) [13, 28, 29]. In the present, study we demonstrate the hepatoprotective effects of remote ischemic conditioning in the recipient through numerous parameters known to be highly relevant in IRI and RIC, in an arterialized rat liver transplantation model using multiple survival time points and various RIC protocols.

**Table 1:** Experimental and clinical studies with remote ischemic conditioning and liver transplantation (search date: 26^th^ of January 2018).

Author	Study type	Species and Strain	Modell or Patient group	Sample size	RIC	CIT and Solution	Time points	Outcome and Conclusion
***Experimental studies***								
**Czigany et al. (present study)**	Experimental	Rat, Lewis, inbred	Arterialized whole graft OLT	144 rats, 72 OLTs	in the recipient, 4x5 min IR, before hepatectomy, after reperfusion, infrarenal aorta	8 h, HTK	1, 3, 24,168 h	“RIC might confer potent protection against the detrimental effects of IR injury including tissue damage, apoptosis, graft circulation, inflammation, tissue energetic status in OLT. HO-1 overexpression might play an orchestrating role in RIC mediated organ protection.”
**Wang et al. [28]**	Experimental	Rat, Sprague-Dawley, outbred	Non-arterialized 30% partial LT	Not clearly described in text, n = 3 OLTs/group/time point, estimated 24 OLTs?	in the donor, 4x5 min IR, lower limb tourniquet	60 min, Ringer	2, 6, 12, 24 h	“RIC can protect liver cells against ischemia reperfusion injury in the small grafts and enhance liver regeneration. Interleukin-6 may be a critical mediator in the stimulatory effect on liver cell regeneration.”
**Jia et al. [13]**	Experimental	Rat, Sprague-Dawley, outbred	Non-arterialized whole graft OLT	48 rats, 24 OLTs	in the recipient, 3x5 min, 3x10 min, 3x1 min IR, immediately at the onset of anhepatic phase, lower limb tourniquet	40 min, Saline	3 h	“The RIC 5minx3 algorithm seemed to be more efficient to alleviate IR injury of the liver graft in both functional and morphological categories, which due to its anti-oxidative, anti-inflammation activities and activating PI3K Akt pathway.”
**Liang et al. [29]**	Experimental	Rat, Sprague-Dawley, outbred	Non-arterialized whole graft OLT	24 rats,12 OLTs	in the recipient, 3x5 min IR, immediately at the onset of anhepatic phase, lower limb tourniquet	45 min, Saline	3 h	“In conclusion, we used an RIC model and confirmed that IR injury was prevented by altered organelles’ Ca2+ status via the Mfn2-MICUs axis”
***Clinical studies***								
**Robertson et al. [55]**	Clinical, Randomized controlled pilot study	Human subjects	Deceased donor whole graft OLT	40 OLTs randomized into RIC or sham	in the recipient, 3x5 min IR, before surgery, left leg pneumatic tourniquet	470±140; 455±157 min UW	90 days follow-up	“RIC is acceptable and safe in liver transplant recipients. This study has not demonstrated evidence of a reduction in short-term measures of IR injury. Longer follow up will be required and consideration of an altered protocol.”
**Koneru et al. (NCT02635347)**	Clinical, Phase I Feasibility and Safety Study	Human subjects	Deceased donor whole graft OLT	50 OLTs single arm enrolment	in the recipient, 3x5 min IR, before hepatectomy and repeated on the initial four post-transplant days, lower limb tourniquet	Not stated	90 days follow-up	Not-applicable, ongoing study

Literature search (PubMed, clinicaltrials.gov) resulted in five relevant studies in liver transplantation and RIC. The other three experimental works (Wang et al, Jia et al., Liang et al.) are studies with lower sample sizes and with clinically less relevant models (non-arterialized graft, not using clinically relevant organ preservation solutions or preservation times, short or only single follow-up time points) or using RIC only as a partial focus of their experiments. According to our knowledge, our study is the most comprehensive experimental work so far investigating the effects of RIC in IR injury following OLT using clinically relevant experimental design, different RIC protocols and multiple follow-up time points. Currently one ongoing Phase I clinical study can be identified from the State University of New Jersey, testing the feasibility and safety of RIC in OLT. A recently published pilot study of Robertson et al. showed feasibility and safety of RIC in OLT without significant benefit during the short term follow-up period used.

Abbreviations: RIC-remote ischemic conditioning; OLT-orthotopic liver transplantation; IR-ischemia reperfusion; PI3K-phosphoinositide 3-kinase; Mfn2-mitofusin 2; MICUs-mitochondrial Ca2+ uptake proteins; HTK-Histidine-tryptophan-ketoglutarate solution; UW-University of Wisconsin solution

Impairment in graft macro- and microcirculation is a key element in ischemia-reperfusion following liver transplantation. Different mechanisms are contributing to the post-ischemic microcirculatory failure such as endothelial cell swelling, neutrophil stasis, sludges and formation of microthromboses [30]. We monitored liver graft microcirculation and red blood cell velocity with an O2C device. Remote conditioning resulted in preserved microcirculation and velocity during the early phase of graft reperfusion. Portal venous flow was likewise improved with the use of remote conditioning and showed similar characteristics, however, the difference between RIC 1 and Control groups became significant after 24 hours of reperfusion. However, due to the complex regulation of hepatic macro- and microcirculation in IR injury, these observations are probably rather secondary manifestations of a more complex mechanistic picture which has not been addressed within this single study [31]. Positive effects of remote ischemic conditioning on target organ circulation were confirmed in various IR injury models [10, 32, 33].

In a prospective clinical study, Puhl et al. demonstrated an inverse relationship between the deteriorating acute post-transplant microcirculation and increased early hepatocellular damage in recipients of cadaveric liver grafts [34]. Our results are in line with the aforementioned clinical findings. In our study the peak of hepatocellular injury was observed at 24 hours of reperfusion according to the histopathological scoring. At this time point, total injury score was found to be significantly higher in the Control group when compared to the RIC 1 group. Serum AST, ALT and LDH release showed a good association with microscopic damage throughout the experiments. The potent infarct/necrosis size limiting effects of RIC (necrosis reduction even by 53–80%, depending on the research setting) was confirmed by others [15, 35, 36]. In a previous study we have demonstrated a significant reduction in tissue injury following the use of remote ischemic conditioning in a rat model of 60 min warm ischemia and 24 hours of reperfusion, measured quantitatively via automated histological image analysis [15].

The deterioration of tissue energetic status is also a crucial factor in IR induced liver injury [30]. Reduction of tissue ATP content results in disturbed active ion transport, thus contributing to cellular swelling and microcirculatory failure [30]. Furthermore, the presence of ATP is also determinative concerning the form of cell-death during IR injury [37]. Previous findings are suggesting the positive effect of various pharmacological and ischemic conditioning approaches on tissue energetic status [38–40]. Our data show a positive tendency in tissue ATP levels during reperfusion with the application of remote conditioning. After 3 hours of reperfusion significantly higher ATP levels were found in the RIC 1 group vs. Control.

It is well documented that imbalance in systemic pro-/anti-inflammatory processes likewise belongs to the major events in the pathophysiology of liver IR injury [2]. IL-10 has an orchestrating role, during the early phase of liver IR, potently reducing pro-inflammatory cytokine and chemokine production [41]. Pretreatment with exogenous IL-10 could dramatically increase survival of genetically obese mice following 15 min of total hepatic ischemia and 24 hours of reperfusion [41]. We found significantly elevated serum IL-10 levels in the remote conditioning groups compared with the Control group after 1 hour of reperfusion. Similar findings were obtained from a previous study in which remote ischemic conditioning induced a significant elevation in serum IL-10 levels in a murine sepsis model which was associated with reduced inflammatory responses and better survival [42]. MCP-1 has multiple effects (chemokine for monocytes, contributing to apoptosis and biliary fibrosis) [43]. In a previous study significantly higher MCP-1 levels were found on the first postoperative day in patients who developed early graft dysfunction within the first week post-transplantation [44]. In our model a prominent increase was detected in serum MCP-1 levels at the 1^st^ postoperative day with slightly lower levels after RIC, however, no significant difference could be shown between the RIC groups and the Control group.

Malondialdehyde is an end-product of the hazardous lipid peroxidation occurring during liver IR, due to the extensive oxidative stress [45]. Despite the graphically conspicuous tendency between groups throughout the observation period, no significant difference could be detected concerning liver MDA levels in the present study. This might be attributed to the relatively high standard deviations observed. Although, here we can find some contradiction to the results of Jia et el., who could demonstrate a significant reduction in tissue MDA levels using a different RIC protocol in a rat model of liver transplantation [13], the radically different transplantation models used and circumstances of the study might provide an explanation for this phenomenon.

HO-1 is a stress protein (Hsp32) and key enzyme of heme catabolism. Both HO-1 itself and the products of heme catabolism (e.g. carbon monoxide, biliverdin) play a role in cytoprotection against IR injury via immunomodulatory, anti-apoptotic, and vasoactive properties [46]. Besides its key anti-ischemic regulator role, its important anti-rejection features have also been demonstrated in liver transplantation [46]. In a comprehensive experimental study of Wang et al., using a murine warm IR injury model, the investigators could demonstrate the potent HO-1 inducing effects of remote ischemic conditioning [47]. The authors concluded that RIC induced up-regulation of HO-1 may act as a key waypoint in autophagy and apoptosis, triggering signal kinase pathways to induce autophagy, and then devour the damaged mitochondria to inhibit apoptosis, and eventually to protect hepatic cells from IR injury [47].

Accordingly, we could find an approximately 15-20-fold upregulation of liver tissue HO-1 encoding mRNA compared to healthy animals after 3 hours of liver transplantation in our model. HO-1 was further upregulated after the application of RIC resulting in significant differences between the RIC 1 and Control groups. Additionally, the potential anti apoptotic properties of HO-1 were supported by our findings with the pBAD/BAD ratio. The phosphorylation of the BAD protein and a consequentially higher pBAD/BAD ratio has an anti-apoptotic effect [48, 49]. In our study an increased pBAD/BAD ratio after 1 hour of reperfusion showed a close association with the observed reduction in the numbers of positive cells in TUNEL immunohistochemistry after 3 hours. During the later phase of reperfusion necrosis became the dominating form of cell death. The apoptosis limiting effects of RIC has been previously demonstrated in various studies [29, 50–52].

In the present model the RIC 1 protocol seemed to be more efficient in reducing tissue injury, improving circulation parameters and preserving tissue energetic status. A certain timing factor of ischemic conditioning and target organ IR injury (before, during, or after) has already been described by others [53, 54]. It can be assumed that an earlier intervention in the time course of IR injury, like in case of the RIC 1 protocol, might have a more potent effect than a postconditioning-like approach (RIC 2). An earlier report has suggested that remote preconditioning is more dependent on neural mechanisms, which are seemingly needed to be activated as soon as possible during the course of ischemia, meanwhile remote postconditioning relies rather on humoral responses [16]. In the present study an earlier preconditioning-like approach could exert a more potent effect on the denervated donor liver, therefore this hypothesis could not be confirmed and our results rather support the co-dependence of the neuro/humoral pathways. Certain time-dependent differences in signal transduction have also been suggested by Hausenloy et. al. [36].

In conclusion, the present study shows the positive effects of remote ischemic conditioning in a clinically relevant experimental model of rat liver transplantation. RIC seemed to be a feasible method which could potently reduce tissue injury, apoptosis, improve graft circulation, positively influence inflammatory cytokine expression, and preserve tissue ATP levels, parallel to the striking upregulation of tissue HO-1 in our setting. An earlier intervention using the RIC 1 protocol was more effective than remote conditioning after graft reperfusion. Conditioning protocol has been adopted from our previous studies using warm IRI models and partial hepatectomy [10, 15]. One limitation of the present work is that no attempt was made to use repetitive conditioning or different RIC cycles to exploit an eventual additive effect of a such protocol. Further limiting factors are the lack of a repetitive sample collection protocol from the same animal and therefore limited follow-up time points, due to animal welfare and 3R (refining severity as well as reducing the numbers of animals) considerations. Although, we acknowledge that our study could not reveal deep mechanistic aspects of the RIC procedure, based on the present findings, we can conclude that intact innervation of the target organ is not essential for the protective effects of RIC in OLT. The exact role and co-dependence of the neuro/humoral mechanisms of RIC in the special scenario of solid organ transplantation and the regulator role of HO-1 are needed to be further elucidated. More detailed exploration of the mechanistic dissimilarities between differently timed remote conditioning interventions as well as functional investigations addressing the effects of RIC on survival and post-transplant liver function would be of interest for basic and translational research.
